# Early screen exposure and its association with motor affordances in the home environment of Brazilian preterm and full-term infants

**DOI:** 10.3389/fped.2026.1833960

**Published:** 2026-07-17

**Authors:** Amanda dos S. Erhardt, André Luis Ferreira de Meireles, Natália Alves Menegol, Anilsa Suraya Gaspar Francisco Gulonda, Bruna Frata, Dayane Montemezzo, Luciana Sayuri Sanada

**Affiliations:** Programa de pós-graduação em Fisioterapia, Universidade do Estado de Santa Catarina, Florianópolis, SC, Brazil

**Keywords:** child, home opportunities, motor development, screen time, technology

## Abstract

**Introduction:**

Although research on screen exposure in infants has grown, little is known about the differences in screen time exposure between term and preterm infants, and how these differences may influence motor development. Therefore, the objectives of the present study were to characterize and compare screen exposure in preterm and full-term infants from a developing country, and to investigate associations between family sociodemographic characteristics, motor development, and home environment opportunities in both groups.

**Methods:**

We analysed data from a cross-sectional study conducted with parents of 80 full-term and preterm infants. The Alberta Infant Motor Scale was used to assess motor development. Parents completed the Affordances in the Home Environment for Motor Development—Infant Scale (AHEMD-IS) and a self-report questionnaire on sociodemographics, their infant's health, and the exposure of both infants and parents to digital screens.

**Results:**

Although no statistically significant difference was observed, the median (interquartile range [IQR]) screen exposure of full-term and preterm infants was 3.5 [7] and 0 [7] hours per week, respectively. Preterm infants' parents watch television longer 17.5 [IQR] than full-term infants' parents 12 [IQR]. There was a significant correlation between the number of hours that parents watch television and the exposure of preterm infants to screens (*rho* = 0.583; *p* = 0.047). There was an association between family income, and AHEMD-IS (*rho* = 0.581; *p* = 0.047).

**Discussion:**

The findings of the present study demonstrated the importance of providing parents with information about the risks associated with excessive screen exposure to infancy, emphasizing that parents play a central role in shaping developmental environment of their children. Parents should be cautioned about the presence of a television playing in the background while infants are at play or present. The present study concluded that, although no statistically significant differences were observed between groups, full-term infants exhibited a trend toward longer screen exposure compared with preterm infants. This study also showed that the time that parents watch TV was associated with their preterm infant's motor development.

## 1 Introduction

In today's digital age, the Internet has become an essential part of human life (1). Excessive or inappropriate screen exposure in children reduces natural play, raising concerns about their physical health, mental well-being, and overall development. It can lead to cognitive deficits, delayed motor skills, reduced communication abilities, social difficulties, and impaired problem-solving skills (1–5). Additionally, exposure to screens during the first years of life can negatively affect parent-child communication and play interactions; it can even affect peer play engagement (6–8). It is likely and plausible to imagine that these negative effects are also present in preterm infants exposed to screens, although we still have no evidence on the effects in this population. Vohr et al. (9) followed 414 extremely premature children (born at less than 28 weeks) in a cohort study and found that high screen time (>2 h/day) was associated with lower total IQ and greater deficits in executive functions, such as metacognition, global executive function, inhibition, and attention at 6 and 7 year (9).

One way to mitigate the potential negative effects of screen exposure on babies is to modulate the environments they are exposed to—both the attitudinal environment, through relationships with parents and immediate family, and the physical environment, by providing opportunities to explore spaces, toys, and objects that support their development (10–12). The environmental factors most strongly associated with hindering healthy infant development are unfavorable socioeconomic conditions and low parental education levels, which increase children's vulnerability to malnutrition, social deprivation, and educational disadvantage (13, 14, 44).

Although research on screen exposure in infants has grown, little is known about the differences in screen time (ST) exposure between term and preterm infants, and how these differences may influence motor development or in-home opportunities. Therefore, the objectives of the present study were to characterize and compare screen exposure in preterm and full-term infants from a developing country, and to investigate associations between family sociodemographic characteristics, motor development, and home environment opportunities in both groups. We hypothesize that preterm infants are exposed to fewer opportunities involving a variety of toys and diverse environments, and to higher amounts of ST.

## 2 Materials and methods

This is a cross-sectional, descriptive, and observational study. The study was approved by the Human Research Ethics Committee (number 3.889.927), and it is in accordance with the Declaration of Helsinki.

Participants were selected using a non-probability purposive sampling approach. The inclusion criteria encompassed parents and their full-term infants (≥37 weeks of gestational age [GA]) or preterm infants (<37 weeks of GA); of both sexes; with a chronological or corrected age between 0 and 15 months. Infants with malformations, diagnosed or suspected genetic syndromes, or with congenital infections were excluded. The sample was divided into two groups: the full-term infant group (TG) and the preterm infant group (PG).

A self-surveyed questionnaire for sample characterization was applied to the parents of the infants. This questionnaire included demographic information, birth related details, and active screen exposure [television (TV), computer, and tablet/smartphone]. A full description of questionnaire are displayed in Supplementary Material 1.

The Affordances in the Home Environment for Motor Development—Infant Scale (AHEMD-IS) is designed to assess the quantity and quality of affordances in the home environment (15). This instrument consists of 35 questions that are easy to understand and quickly answered by parents (16). The 35 items of the questionnaire are subdivided into four dimensions: (1) Physical space, (2) Variety of stimulation, (3) Gross motor function toys, and (4) Fine motor function toys. The total score of the instrument is obtained by the sum of the scores of each dimension. This score was divided based on the indexes and classified for each dimension as less than adequate, moderately adequate, adequate, and excellent for the age group evaluated (15).

Additionally, motor development was assessed by Alberta Infant Motor Scale (AIMS), which is an observational tool, with high inter-rater reliability (17). It contains 58 items divided into prone, supine, sitting, and standing subscales. The total score is the sum of the criteria observed in each subscale (0 to 58 points), and after application, they can be transformed into percentiles (17). The AIMS was measured through AIMS by previously trained evaluators (ASE; NAM; ASGFG).

The collected data were coded and entered into the Statistical Package for the Social Sciences (SPSS; IBM Corp., Armonk, NY, USA, version 20), with the significance level set at *p* < 0.05. Data normality was assessed using the Kolmogorov–Smirnov test. For comparison between term and preterm, the Mann–Whitney test was used, and the effect size was calculated by the formula $\left(\eta^{2}=\frac{z^{2}}{N};\;z=\frac{\left[U\;-\frac{\left(\eta 1\;\times \eta 2\right)}{2}\;-\;0.5\right]}{\sqrt{(\eta 1\times \eta 2)\;\times \;\frac{\left(\eta 1+\eta 2+1\right)}{12}}}\right)$ (18, 19). For categorical variables, the Chi-Square test was used, and a *post hoc* analysis was performed in accordance with the method proposed by MacDonald and Gardner to adjust the *α* level (*α*_corr_) according to the number of cells. Significant associations were identified based on adjusted residuals and corrected *p*-values (20).

To examine the correlation between the parental ST of parents, infant ST, family income, parental education level, AIMS percentile, and AHEMD-IS classifications, Spearman's correlation test was applied to each group.

## 3 Results

Eighty infants were evaluated, 68 (85%) corresponding to TG. The median [interquartile range—IQR] of the age of the TG was 5.8 [5.6] months, while the median of PG was 4.14 [3] months, considering the corrected age. The median of the GA of the TG was 39.4 [1.3] weeks, while the PG was 33 [5.7] weeks. The sample characterization is shown in Table 1. There was no significant difference between groups in sociodemographic data and parents' level of education.

**Table 1:** Description of neonatal and sociodemographic characteristics. Data showed in absolute and relative frequencies.

Variables	TG (*n* = 68)	PG (*n* = 12)	Total sample (*n* = 80)	*p* value
Corrected Age (months)^b^				
0 to ≤3	6 (8.8%)	5 (41.7%)	11 (13.8%)	.083
>3 to ≤6	32 (47.0%)	3 (25%)	35 (43.8%)	
>6 to ≤9	12 (17.6%)	2 (16.7%)	14 (17.5%)	
>9 to ≤12	8 (11.8%)	1 (8.3%)	9 (11.2%)	
>12 to ≤15	7 (10.3%)	0 (0.0%)	7 (8.8%)	
>15 to ≤18	3 (4.4%)	1 (8.3%)	4 (5.0%)	
Gender^a^				
Male	38 (55.9%)	5 (41.7%)	43 (53.7%)	.363
Female	30 (44.1%)	7 (58.3%)	37 (46.2%)	
Type of delivery^b^				
Normal	27 (39.7%)	2 (16.7%)	29 (36.2%)	.203
Induced	4 (5.9%)	0 (0.0%)	4 (5.0%)	
Caesarean	35 (51.5%)	10 (83.3%)	45 (56.2%)	
Unreported	2 (2.9%)	0 (0.0%)	2 (2.5%)	
Birth complications^b^				
None	57 (83.8%)	10 (83.3%)	67 (83.8%)	.361
Fetal distress	6 (8.8%)	0 (0.0%)	6 (7.5%)	
Others	5 (7.4%)	2 (16.7%)	7 (8.8%)	
Hospitalization in NICU^a^				
Yes	5 (7.4%)	9 (75.0%)	14 (17.5%)	<.001
No	63 (92.6%)	3 (25.0%)	66 (82.5%)	
Household income^b^				
Less than R$ 1,900/month	12 (17.6%)	3 (25.0%)	15 (18.8%)	.403
R$ 1,900 to R$ 3,800/month	18 (26.5%)	2 (16.7%)	20 (25.0%)	
R$ 3,800 to R$ 9,540/month	22 (32.4%)	6 (50%)	28 (35.0%)	
R$ 9,540 to R$ 19,080/month	15 (22.1%)	0 (0.0%)	15 (18.8%)	
More than R$ 19,080/month	1 (1.5%)	1 (8.3%)	2 (2.5%)	
Father's education^b^				
No formal education or incomplete elementary school	4 (5.9%)	1 (8.3%)	5 (6.2%)	1.000
Elementary school	1 (1.5%)	0 (0.0%)	1 (1.2%)	
High school	21 (30.9%)	4 (33.3%)	25 (31.2%)	
Graduated	35 (51.5%)	6 (50%)	41 (51.2%)	
Master's or doctor's degree	7 (10.3%)	1 (8.3%)	8 (10.0%)	
Mother's education^b^				
No formal education or incomplete elementary school	0 (0.0%)	1 (8.3%)	1 (1.2%)	.356
Elementary school	4 (5.9%)	0 (0.0%)	4 (5.0%)	
High school	14 (20.6%)	3 (25%)	17 (21.2%)	
Graduated	41 (60.3%)	7 (58.3%)	48 (60%)	
Master's or doctor's degree	9 (13.2%)	1 (8.3%)	10 (12.5%)	
Area of residence^a^				
Urban	66 (97.1%)	12 (100%)	78 (97.5%)	1.000
Rural	2 (2.9%)	0 (0.0%)	2 (2.5%)	
Type of residence^b^				
House	20 (29.4%)	4 (33.3%)	24 (30%)	
Apartment	46 (67.6%)	8 (66.7%)	54 (67.5%)	1.000
Other	1 (1.5%)	0 (0%)	1 (1.25%)	
Unreported	1 (1.5%)	0 (0%)	1 (1.25%)	

TG, term group; PG, preterm group; NICU, Neonatal Intensive Care Unit.

a indicates that the Fisher test was applied to compare the groups.

b indicates that the chi-square test was applied to compare the groups.

Table 2 presents data on screen exposure among infants and their parents. A statistical comparison between the groups revealed a significant difference in the number of hours parents spent watching TV (*p* = 0.031), indicating that parents in the PG watched TV for longer than those in the TG. Table 3 shows the absolute and relative frequencies of the types of programs watched by infants, with no significant differences between groups.

**Table 2:** Time of exposure to screens by infants and their parents, presented in hours per week.

Variables	TG	PG	*p*-value	*η*²
	Median (Min-Max)	Median (Min-Max)		
TV	3.5 (0–28)	0.45 (0–21)	.458	.01
Tablet/cell phone	0 (0–7)	0 (0–0)	.461	.01
Total Infant's Screen Time	3.5 (0–28)	0.45 (0–21)	.399	.01
TV viewing time of parents	12 (0–35)	17.5 (7–35)	.031^a^	.06
Total Parents' Screen Time	8.5 (0–56)	17 (0–21)	.753	.01

TG, term group; PG, preterm group; Min, minimum; Max, maximum.

a Data showing significant differences between the groups (*p* ≤ 0.05). Comparison between groups using the Mann–Whitney *U* Test; The effect size (*η*²) was calculated by the formula $\left(\eta^{2}=\frac{z^{2}}{N};\;z=\frac{\left[U\;-\frac{\left(\eta 1\;\times \eta 2\right)}{2}\;-\;0.5\right]}{\sqrt{(\eta 1\times \eta 2)\;\times \;\frac{\left(\eta 1+\eta 2+1\right)}{12}}}\right)$.

**Table 3:** Absolute and relative frequencies of the types of programs watched by infants. Parents could choose more than one type of answer.

Type of program watched by infants^a^	TG (*n* = 68)	PG (*n* = 12)	Total sample (*n* = 80)	*p* value
Children's television programming^b^	40 (58.8%)	6 (50.0%)	46 (57.7%)	.396
Adult television program^c^	9 (13.2%)	1 (8.3%)	10 (12.5%)	.536
Does not watch TV	24 (35.3%)	6 (50%)	30 (37.5%)	.351
Fed in front turned on screen	7 (10.3%)	2 (16.7%)	9 (11.25%)	.617

TG, term group; PG, preterm group;

a Parents were allowed to select more than one response;

b included cartoon, musical, movies;

c included TV serie, soap opera, newcast.

The Fisher test was applied to compare the groups for all variables.

The parents of 27 infants in the TG (39.7%) and 2 infants in the PG (16.7%) believe that TV contributes to their child's education. Additionally, 16 TG parents (23.5%) and 2 PG parents (16.7%) believe that tablet or mobile phone video games support infant development, whereas 53 TG parents (77.9%) of and 10 PG parents (83.3%) do not share this belief. Two parents (2.5%), one from each group, did not express an opinion. No significant differences between groups (Table 4).

**Table 4:** Absolute and relative frequencies of the types of TV programs that parents believe enhance infant education, and areas of infant development that parents believe are improved by games on tablet/cell phones.

Variables	TG (*n* = 68)	PG (*n* = 12)	Total sample (*n* = 80)	*p* value
TV programs^a^				
Educational cartoons	21 (30.9%)	2 (16.7%)	23 (28.8%)	.493
Musical cartoons	10 (14.7%)	1 (8.3%)	11 (13.8%)	1.000
Others	4 (5.9%)	1 (8.3%)	5 (6.3%)	.571
Believe that TV do not improve infant education	41 (60.3%)	10 (83.3%)	51 (63.8%)	.194
Believe that games do not improve	53 (77.9%)	10 (83.3%)	63 (78.8%)	1.000
Development Area^a^				
Sensoriomotor	5 (7.4%)	0 (0.0%)	5 (6.3%)	.434
Cognition	12 (17.6%)	1 (8.3%)	(16.3%)	.377
Do not know	1 (1.5%)	1 (8.3%)	2 (2.5%)	.279

TG, term group; PG, preterm group;

a Parents were allowed to select more than one response.

The Fisher test was applied to compare the groups for all variables.

Table 5 presents the absolute and relative frequencies of motor development, categorized by percentiles curves according to the AIMS scores. Twelve (17.6%) full-term infants and one (8.3%) preterm infant had a percentile below the 5th percentile curve. No significant difference was observed between the groups.

**Table 5:** Absolute and relative frequencies of motor development assessment according to Alberta infant motor scale percentile.

	TG (*n* = 68)	PG (*n* = 12)	Total sample (*n* = 80)	*p* value	Adjuted *p* value
Percentile					
<5%	12 (17.6%)	1 (8.3%)	13 (16.2%)	.310^a^	.423
5%	2 (2.9%)	1 (8.3%)	3 (3.8%)		.368
>5 to 10%	3 (4.4%)	0 (0.0%)	3 (3.8%)		.484
>10 to 25%	11 (16.2%)	2 (16.7%)	13 (16.2%)		1.000
>25 to 50%	20 (29.4%)	1 (8.3%)	21 (26.2%)		.134
>50 to 75%	11 (16.2%)	3 (25%)	14 (17.5%)		.484
>75 to 90%	9 (13.2%)	4 (33.3%)	13 (16.2%)		.089

TG, term group; PG, preterm group.

a Comparison between groups using the Chi-Square Test, where *α*_corr_ = 0.003125.

Table 6 displays the data of the AHEMD-IS. A significant difference was found between the TG and PG in the variety of stimulation score (*p* = 0.022), as well as in the total score of the AHEMD-IS (*p* = 0.023). No significant differences were observed between the TG and PG in the other dimensions.

**Table 6:** Scores for the dimensions and total classification of the affordances in the home environment for motor development—infant scale, presented as median and interquartile range. The total classification is shown in absolute and relative frequencies.

Domain	TG (*n* = 68)	PG (*n* = 12)	Total sample (*n* = 80)	*p* value	*η*²
Physical Space	5 [4]	3.5 [5]	5 [5]	.102	.03
Variety of Stimulation	13.5 [4]	11.5 [5]	13 [4]	.022^a^	.07
Gross-Motor Toys	6 [3]	6 [2]	6 [3]	.239	.02
Fine-Motor Toys	4.5 [4]	3 [4]	4 [3]	.071	.04
Total Score	28 [10]	21.5 [13]	27.5 [11]	.023^a^	.06

Total Classification	TG (*n* = 68)	PG (*n* = 12)	Total sample (*n* = 80)	*p* value	Adjuted *p* value
Less than adequate	7 (10.3%)	5 (41.7%)	12 (15%)	.069*	.005^b^
Moderately adequate	13 (19.1%)	2 (16.7%)	15 (18.8%)		.841
Adequate	17 (25%)	2 (16.7%)	19 (23.8%)		.548
Excellent	31 (45.6%)	3 (25%)	34 (42.5%)		.193

TG, term group; PG, preterm group. Comparison between groups using the Mann–Whitney *U* Test; The effect size (*η*²) was calculated by the formula $\left(\eta^{2}=\frac{z^{2}}{N};\;z=\frac{\left[U\;-\frac{\left(\eta 1\;\times \eta 2\right)}{2}\;-\;0.5\right]}{\sqrt{(\eta 1\times \eta 2)\;\times \;\frac{\left(\eta 1+\eta 2+1\right)}{12}}}\right)$. The comparison between groups on total classification was applied the Chi-square test, where *α*_corr_ = 0.00625.

a Data showing significant differences between the groups (*p* ≤ 0.05).

b Data showing significant differences between the groups (adjusted *p* ≤ 0.00625).

There was no correlation between biological variables and motor development, screen exposure, or home opportunities in both groups. Also, motor development was not correlated with screen exposure in the TG (*p* = 0.392, *rho* = −0.105) or the PG (*p* = 0.372, *rho* = −0.284).

Regarding the TG, the following correlations were observed: a regular correlation of family income and the classification of fine motor toys (*p* = 0.015, *rho* = 0.295), and the total classification of the AHEMD-IS (*p* = 0.020, *rho* = 0.281). Also, regular correlation of the mother's education and the classification of fine motor toys (*p* = 0.006, *rho* = 0.331), and the total classification of the AHEMD-IS (*p* = 0.037, *rho* = 0.254). Full details are presented at eTable 1 in Supplementary Material 2. There was no correlation between the hours per week that parents watch TV and the total infants' screen exposure (*p* = 0.987, rho = −0.002).

Concerning the PG, there was a moderate to good correlation among family income, the classification of physical space (*p* = 0.046, *rho*=0.585), and the total classification of the AHEMD-IS (*p* = 0.047, *rho* = 0.581); a moderate to a good correlation of the mother's education, the classification of gross motor skills toys (*p* = 0.016, *rho* = 0.675), and total AHEMD-IS classification (*p* = 0.047, *rho* = 0.582). Full details are presented at eTable 1 in Supplementary Material 2. It was also observed moderate to good correlation between the hours per week that parents watch TV and the total infants' screen exposure (*p* = 0.047, *rho* = 0.583); and a moderate to a good correlation of the hours per week that parents watch TV with the AIMS percentile (*p* = 0.019, *rho* = −0.662).

## 4 Discussion

Over the past decade, numerous studies have examined the impact of screen exposure on children, yet little is known about its effects during the first months of life or the factors driving early exposure—especially in developing countries such as Brazil. In the present study, most infants—both fullterm and preterm—exceeded the recommended limits for ST (21–23). Although no statistically significant differences were observed between groups, the median total weekly screen time was 3.5 h among full-term infants and 0.45 h among preterm infants.

An additional noteworthy finding was that, despite the absence of differences in total parental screen time between groups, parents in the PG reported significantly greater television viewing time. Futheremore, there was a significant correlation between the parental ST and infant ST in PG group. This pattern suggests that the distribution of screen-based activities, rather than the overall volume of screen exposure, may vary according to the caregiving context. Previous studies indicate that parental media practices are shaped not only by individual preferences but also by family routines, caregiving demands, and the availability of cognitive and temporal resources (24–26). In families of preterm infants, the increased burden associated with ongoing health monitoring, developmental concerns, and caregiving responsibilities may favor more passive forms of media consumption, such as television viewing, compared with interactive devices that require greater engagement. Television may also function as a background medium integrated into daily caregiving routines, providing information, companionship, or distraction during prolonged periods spent at home. This interpretation is consistent with previous research indicating that mothers of preterm infants are more likely to adopt controlling interaction styles and exhibit lower levels of emotional involvement during interactions with their infants (45–48).

Another important finding of the present study is that, in addition to exceeding the recommended screen time limits, some infants in both groups were exposed to age-inappropriate content, such as news broadcasts that may include inappropriate language and violent imagery. Studies have demonstrated that exposure to such programs negatively influences development, as they display content unsuitable for infants and lead parents to interfere with socioemotional interactions (27, 28). Furthermore, Vandewater, Bickham, and Lee (29) showed a negative relationship between TV exposure and time spent in creative play among children aged 0 to 2 years (29). Thus, background exposure to TV can negatively influence infant development by reducing opportunities for social, emotional, and physical interaction between parents and their children; this is even more relevant for children at biological risk, such as those born preterm.

Chiu et al. (30), who reported that parental behavior regarding television played a crucial role in shaping children's screen habits, with children potentially adopting TV-related behaviors similar to those of their parents. Similarly, Brushe et al. (6) found that parental ST may be directly associated with the time children spend in front of screens at 12 and 36 months of age, with negative implications for language development and family bonding. Additionally, a cohort study evaluating the effects of screen exposure in 414 children born preterm, aged 6–7 years, found that those exposed to more than two hours of ST per day had lower IQ scores and greater deficits in executive functioning compared to children with less than two hours of daily ST (9). Although there is still limited information on the effects of screen exposure on preterm infants and its long-term consequences, it is plausible that preterm infants—who already face more developmental challenges—may be more adversely affected than full-term infants.

The AHEMD-IS score for variety of stimulation in the physical space was also lower in the PG, indicating that, in addition to exceeding recommended ST these infants had fewer opportunities for stimulation in their physical environments, which may have developmental repercussions. From this perspective, Kirkorian et al. (28) showed that background TV not only reduces the quantity and quality parent-child interactions but also decrease active parental involvement and the capacity to provide verbal stimulation. Another study, involving 89 infants assessed at 12 and 24 months, demonstrated that children's ST was positively associated with body fat index and negatively associated with development outcomes assessed by the Balyey-III scale (3). Although our study found no correlations between motor development and screen exposure in infants, it is important to consider that sedentary behavior associated with ST may reduce opportunities for physical activity provided by parents (31).

Some studies investigating parental characteristics and sociodemographic factors have report a relationship between higher education and income levels and lower ST exposure for children (6, 32). Both groups in our study showed significant correlations between parental education and AHEMD-IS classifications, which may be associated with the parent's greater access to information when they have higher education levels. Defilipo et al. (33) showed that both maternal and paternal education are associated with the quantity of developmental opportunities at home. Another study found that higher maternal education predicts better quality and organization of the physical environment and a greater variety of daily stimulation, including the availability of appropriate materials and toys for infants. Furthermore, as Jeong et al. (34) discussed, children in developing countries are more likely to be exposed to multiple risk factors for poor development outcome.

Regarding life-at-home opportunities, the present study identified a significant correlation between family income and the classifications of physical space, fine motor skills, and total AHEMD-IS classification. This result reinforces the idea that lower socioeconomic levels are directly associated with environments offering fewer varieties of stimulation and developmental opportunities (33, 35–37). Given the importance of the family socioeconomic status in infants' development, studies have shown that the environment and available opportunities directly impact language skills, learning, memory, executive functions, cognition, and emotional regulation (13, 38–40).

As for the types of programs parents perceived as beneficial, educational cartoons stood out. However, evidence suggests that regardless of the content viewed by infants, verbal interaction between parents and infants is restricted during these moments, and becomes even less frequent when exposure involves educational content (41, 42). Moreover, a study reported a negative association between viewing baby-specific DVDs/videos and vocabulary acquisition in infants aged 8–16 months. These videos often contain minimal dialogue, short scenes, disconnected imagery, and visually stimulating but linguistically ambiguous events (43).

Although the present study provides valuable insights for guiding future policies on screen exposure in infants from developing countries, some limitations must be highlighted. The questionnaire applied did not distinguish between active screen use by infants and background television exposure, where no direct infant engagement with the screen occurred. Furthermore, it did not assess parental work-related pressures or the family's daily routines, which may represent relevant contextual factors influencing the study outcomes. The assessment relied on parental self-reported screen time, which may have introduced bias due to inaccurate reporting. Additionally, the disparity in sample sizes between full-term and preterm infants may have influenced certain statistical results. The relatively small number of preterm infants included in the study limits the generalizability of the results; consequently, all findings should be regarded as exploratory. Moreover the cross-sectional design did not allow for an analysis of the impact of screen exposure on motor development over time. The statistical analysis was limited to simple correlations between screen use and motor development, which may have influenced the findings.

Future studies should include groups with more comparable sample sizes and adopt longitudinal designs to better assess the long-term effects of screen exposure on motor development in infants, as well as potential differences in exposure between full-term and preterm infants.

The findings of the present study demonstrated the importance of providing parents with information about the risks associated with excessive screen exposure to infancy, emphasizing that parents play a central role in shaping developmental environment of their children. Parents should be cautioned about the presence of a television playing in the background while infants are at play or present. It also important to highlight that parental ST may affect the quality of parent-infant interactions, potentially impacting development—especially in preterm infants.

The present study concluded that, although no statistically significant differences were observed between groups, full-term infants exhibited a trend toward longer screen exposure compared with preterm infants. Also, the time parents spend watching TV, family income, and parents' education negatively were associated with life-at-home opportunities available to infants. This study also showed that the time that parents watch TV was associated with their preterm infant's motor development.

## Data Availability

The raw data supporting the conclusions of this article will be made available by the authors, without undue reservation.
