# Supplementary material for: Early screen exposure and its association with motor affordances in the home environment of Brazilian preterm and full-term infants
Source: Front Pediatr. 2026 Jul 17;14:1833960. doi: 10.3389/fped.2026.1833960 (PMC13424202; doi:10.3389/fped.2026.1833960)
Supplement: Supplementary file 1 [file Supplementaryfile1.docx]

**SUPPLEMENTARY MATERIAL 1 - PARENT OR LEGAL GUARDIAN QUESTIONNAIRE**

**Identification Data**

Who is answering the questionnaire? (Relationship to the child) ( ) Mother ( ) Father ( ) Other guardian

Age of the father, mother, or guardian: ________

Do you have other children? ( ) No ( ) Yes. How many and what are their ages? __________

Do you practice physical activities? [ ] No [ ] Yes

If yes, how many times per week? ( ) Once ( ) Twice ( ) Three or more times per week

**Gestational Information (related to the Baby’s Pregnancy)**

Gender: [ ] Male [ ] Female

Date of birth: ***_______________***  Gestational age at birth (weeks): ______

Birth weight: __________ Height at birth: ________ Head circumference: ______

Apgar score (1st minute): ______ (5th minute): ______ Type of delivery: [ ] Normal [ ] Induced [ ] Cesarean [ ] Forceps

Pregnancy type: [ ] Single [ ] Twins [ ] Triplets

Did you take any prescribed medication during pregnancy? [ ] No [ ] Yes, specify: _______________

Did you experience complications during pregnancy? [ ] No [ ] Bleeding [ ] Hypertension [ ] Diabetes [ ] Infections [ ] Anemia [ ] Other: ______________

Hospitalizations during pregnancy: [ ] None [ ] 1 [ ] 2 [ ] 3 or more

Any complications during delivery? [ ] None [ ] Fetal distress [ ] Cord problems [ ] Other: ________

Did the baby require hospitalization in the NICU? [ ] No [ ] Yes, for what reason? ____________ Duration: __________

Have you observed your baby in a prone position in the NICU? [ ] Yes [ ] No

Did you participate in the Kangaroo Program? [ ] Yes, how many times? ____________ [ ] No

**Child’s Current Health**

Current weight: __________ Current height: ________ Current head circumference: ______

Exclusive breastfeeding: [ ] Yes [ ] No Duration of breastfeeding: ________

Does the child take any medication? [ ] No [ ] Yes, specify: ___________

Does the child have any known medical conditions? [ ] No [ ] Yes, specify: ___________

**Child’s Positioning**

How much time do you spend with your baby daily? ___________

Does your child receive care from babysitters or relatives? [ ] No [ ] Yes, for how long? _____

Are you familiar with the stages of motor development? [ ] No [ ] Yes

Have you received guidance on how to position the baby at home? [ ] No [ ] Yes

Do you have a baby stroller? [ ] No [ ] Yes How many hours per day does the baby stay in the stroller? ____

Do you have a baby car seat? [ ] No [ ] Yes How many hours per day does the baby stay in the car seat? ____

**Child’s Sleep**

Does your child snore while sleeping? [ ] No [ ] Yes

Does your child have difficulty breathing during sleep? [ ] No [ ] Yes

Does your child stop breathing for brief periods during sleep? [ ] No [ ] Yes

Is your child's sleep restless or marked by frequent awakenings? [ ] No [ ] Yes

**Socioeconomic Characteristics**

1. Monthly family income: ( ) Up to 2 minimum wages ( ) 2 to 4 minimum wages ( ) 4 to 10 minimum wages ( ) 10 to 20 minimum wages ( ) Above 20 minimum wages
2. How many people are financially supported by this income? ( ) 1 ( ) 2 ( ) 3 ( ) 4 ( ) 5 or more
3. How many people live in the residence? ( ) 1 ( ) 2 ( ) 3 ( ) 4 ( ) 5 or more
4. Do the child's parents live together? ( ) No ( ) Yes

If separated, who does the child predominantly live with? ( ) Mother ( ) Father ( ) Other. Who? ________________

Does the child maintain contact with the parent they do not live with? ( ) Yes ( ) No. How many times per week? ___________

**Residence Characteristics**

1. Where does your family reside? ( ) Urban Area ( ) Rural Area
2. Type of residence: ( ) Apartment ( ) House ( ) Other, specify: _______________
3. How long has your family lived in this residence? ___________
4. Items available in your home (indicate quantity): [ ] Television [ ] Tablet [ ] Video game

[ ] Computer [ ] Cell phone [ ] Car [ ] Bicycle [ ] Scooter [ ] Skateboard

**Daily Screen Time**

**On a typical day, how much time would you say a child usually spends...**

1. Does the child have a television in their bedroom? ( ) No ( ) Yes

If yes, how many hours does the child usually watch it per day?

( ) 1 hour ( ) 2 hours ( ) 3 hours ( ) 4 hours ( ) 5 hours ( ) Other: ______

Days per week: ( ) 1 ( ) 2 ( ) 3 ( ) 4 ( ) 5 ( ) 6 ( ) 7

1. How many hours does the child spend watching TV?

Per day: ( ) 1 hour ( ) 2 hours ( ) 3 hours ( ) 4 hours ( ) 5 hours ( ) Other: ______

Days per week: ( ) 1 ( ) 2 ( ) 3 ( ) 4 ( ) 5 ( ) 6 ( ) 7

1. What type of programs does the child usually watch?
   ( ) Cartoons ( ) Music ( ) Movies ( ) Series ( ) Soap operas ( ) News programs
   ( ) None of the above
2. How many hours does the parents spend watching TV?

Per day: ( ) 1 hour ( ) 2 hours ( ) 3 hours ( ) 4 hours ( ) 5 hours ( ) Other: ______

Days per week: ( ) 1 ( ) 2 ( ) 3 ( ) 4 ( ) 5 ( ) 6 ( ) 7

1. How many hours does the child spend on videogame?

Per day: ( ) 1 hour ( ) 2 hours ( ) 3 hours ( ) 4 hours ( ) 5 hours ( ) Other: ______

Days per week: ( ) 1 ( ) 2 ( ) 3 ( ) 4 ( ) 5 ( ) 6 ( ) 7

1. How many hours does the child spend on computer?

Per day: ( ) 1 hour ( ) 2 hours ( ) 3 hours ( ) 4 hours ( ) 5 hours ( ) Other: ______

Days per week: ( ) 1 ( ) 2 ( ) 3 ( ) 4 ( ) 5 ( ) 6 ( ) 7

1. How many hours does the child spend on a tablet or smartphone?

Per day: ( ) 1 hour ( ) 2 hours ( ) 3 hours ( ) 4 hours ( ) 5 hours ( ) Other: ______

Days per week: ( ) 1 ( ) 2 ( ) 3 ( ) 4 ( ) 5 ( ) 6 ( ) 7

1. How many hours do parents/caregivers spend on a tablet or smartphone while with the child? Per day: ( ) 1 hour ( ) 2 hours ( ) 3 hours ( ) 4 hours ( ) 5 hours ( ) Other: ______

Days per week: ( ) 1 ( ) 2 ( ) 3 ( ) 4 ( ) 5 ( ) 6 ( ) 7

1. If the child uses a tablet/smartphone, what activities do they engage in?

( ) Games ( ) Cartoon/Movies/Music ( ) Social Media (Facebook, Instagram, etc.)

1. Does the child eat meals while watching TV, movies, or playing? ( ) No ( ) Yes
2. Do you believe television helps in education? ( ) No ( ) Yes.

If yes, what type of program helps? _____________________________________________

_________________________________________________________________________

1. Do you believe games on tablets/smartphones help in child development? ( ) No ( ) Yes.

If yes, which aspect of development? ____________________________________________

_________________________________________________________________________

Comments / Observations:

_________________________________________________________________________________________________________________________________________________________________________________________________________________________________________________________________________________________________________________________________________________________________________________________________
