# Supplementary material for: Early screen exposure and its association with motor affordances in the home environment of Brazilian preterm and full-term infants
Source: Front Pediatr. 2026 Jul 17;14:1833960. doi: 10.3389/fped.2026.1833960 (PMC13424202; doi:10.3389/fped.2026.1833960)
Supplement: Supplementary file 2 [file Supplementaryfile2.docx]

***SUPPLEMENTARY MATERIAL 2***

eTable 1 – Correlation between family characteristics and home opportunities. Data compared by Sperman correlation test.

|  | **TERM** | | |
| --- | --- | --- | --- |
| AHEMD-IS | Family income | Father's education | Mother's education |
| Physical Space | p = .861, rho= - .022 | p = .751, rho= -.039 | p = .816, rho= .029 |
| Variety of Stimulation | p = .132, rho= .184 | p = .066, rho= .224 | p = .412, rho= .101 |
| Gross-Motor Toys | p = .051, rho= .234 | **p = .004, *rho* = 0.345*** | p = .053, rho= .236 |
| Fine-Motor Toys | **p = .015, *rho* = 0.295*** | **p = .017, *rho* =0.288*** | **p=.006, *rho* = 0.331*** |
| Total classification | **p = .020, *rho* = 0.281*** | **p = .004, *rho* = 0.348*** | **p = .037, *rho* = 0.254*** |
| Infant’s total screen time | p = .866, rho= .021 | p = .552, rho= -.073 | p = .169, rho= -.169 |
| Parent’s time on cellphone/tablet | p = .129, rho= .186 | p = .129, rho= .186 | p = .183, rho= .164 |
|  | **PRETERM** | | |
| AHEMD-IS | Family income | Father's education | Mother's education |
| Physical space | **p = .046, *rho*=0.585**** | p = .294, rho= .331 | p = .235, rho= .371 |
| Variety of Stimulation | p = .183, rho= .412 | p = .108, rho= .488 | p = .177, rho= .418 |
| Gross-Motor Toys | p = .078, rho= .527 | p = .144, rho= .448 | **p = .016, *rho* = 0.675**** |
| Fine-Motor Toys | p = .312, rho= .319 | p = .761, rho= .099 | p = .425, rho= .254 |
| Total classification | **p = .047, *rho* = 0.581**** | p = .131, rho= .461 | **p = .047, *rho* = 0.582**** |
| Infant’s total screen time | p = .476, rho= -.228 | p = .175, rho= -.419 | p = .260, rho= -.353 |
| Parent’s time on cellphone/tablet | p = .260, rho= .353 | p = .096, rho= .502 | p = .072, rho= .537 |

Legend: * regular correlation; **moderate to good correlation
